# A Predator from East Africa that Chooses Malaria Vectors as Preferred Prey

**DOI:** 10.1371/journal.pone.0000132

**Published:** 2006-12-27

**Authors:** Ximena J. Nelson, Robert R. Jackson

**Affiliations:** 1 School of Biological Sciences, University of Canterbury, Christchurch, New Zealand; 2 International Centre of Insect Physiology and Ecology (ICIPE), Thomas Odhiambo Campus, Mbita Point, Kenya; University of Exeter, Cornwall Campus, United Kingdom

## Abstract

**Background:**

All vectors of human malaria, a disease responsible for more than one million deaths per year, are female mosquitoes from the genus *Anopheles. Evarcha culicivora* is an East African jumping spider (Salticidae) that feeds indirectly on vertebrate blood by selecting blood-carrying female mosquitoes as preferred prey.

**Methodology/Principal Findings:**

By testing with motionless lures made from mounting dead insects in lifelike posture on cork discs, we show that *E. culicivora* selects *Anopheles* mosquitoes in preference to other mosquitoes and that this predator can identify *Anopheles* by static appearance alone. Tests using active (grooming) virtual mosquitoes rendered in 3-D animation show that *Anopheles*' characteristic resting posture is an important prey-choice cue for *E. culicivora*. Expression of the spider's preference for *Anopheles* varies with the spider's size, varies with its prior feeding condition and is independent of the spider gaining a blood meal.

**Conclusions/Significance:**

This is the first experimental study to show that a predator of any type actively chooses *Anopheles* as preferred prey, suggesting that specialized predators having a role in the biological control of disease vectors is a realistic possibility.

## Introduction

That an East African predator might single out malaria vectors as preferred prey is of considerable interest. Not only is malaria the world's most important insect-borne threat to public health [1], [2], but it is especially in sub-Sahara Africa that *Plasmodium falciparum* and lethal malaria are prevalent [2], [3], [4], [5]. Vectors of human malaria all belong to a particular mosquito genus, *Anopheles* [1], [6], [7]. Here we consider *Evarcha culicivora*, an East African jumping spider [Salticidae]. This species is known only from the vicinity of Lake Victoria in East Africa [8], a region where, even by African standards, the impact of malaria is especially severe [2], [9], [10]. Innate preference for blood-carrying female mosquitoes was shown for all active size classes of *E. culicivora* in an earlier study [11], but finer-grain preference for specifically *Anopheles* was not investigated. Here we show that, when sated, both large and small individuals of *E. culicivora* single out *Anopheles* as their preferred prey, and small juveniles of this predator prefer *Anopheles* even when fasted.

Although often blurred in the literature, distinctions between diet, prey choice and preference [12], [13] are especially important for understanding the biology of *E. culicivora*. A predator's natural diet may suggest hypotheses about prey choice, choice being a behavioral trait driven by preference, and preference being a predator's differential motivation to feed on the different prey types it encounters, but testing these hypotheses depends on experimental data. When quiescent, *E. culicivora* hides in the grass or in other vegetation close to the ground, but feeding individuals venture into more exposed locations, including the inside walls of mosquito-infested houses [8]. By a wide margin, the most abundant mosquito-size insects in these habitats are non-biting midges (Chironomidae and Chaoboridae) [14], known locally as “lake flies”. Yet *E. culicivora*'s natural diet is dominated by female mosquitoes [8] and a subsequent experimental study [11] showed that *E. culicivora* feeds indirectly on vertebrate blood by selecting blood-carrying female mosquitoes as prey.

**Figure 1:**
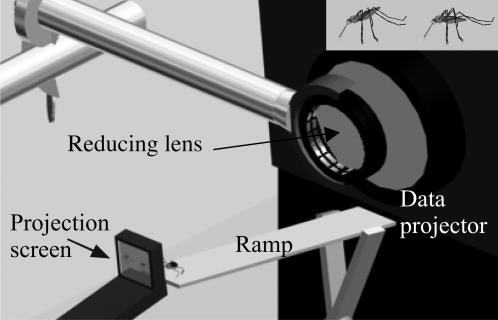
Apparatus for virtual-prey testing. Spider (not to scale) at top of inclined metal ramp, oriented toward one of two side-by-side virtual mosquitoes. Virtual mosquitoes on small screen (projection screen, see enlarged inset in top right corner) positioned in front of higher end of ramp. Images pass from projector lens (connected to computer; main body of data projector and computer not shown) through second lens (for reducing image size) on to screen. Observer point of view: about 150 degrees from the direct light path from the projector through the projection screen; slightly behind the projection screen at a height of approximately 45 degrees. Inset: virtual mosquitoes in *Anopheles* resting posture (left) and in non-*Anopheles* resting posture (right).

For the present study, as in the previous study [11], we take advantage of how the exceptional eyesight of salticids [15], [16], [17], [18], [19] permits testing with motionless lures made from dead prey mounted on cork discs in lifelike posture and also with virtual prey (Fig. 1) rendered with 3-D animation. In another earlier study [20], the small juveniles of *E. culicivora* were shown to adopt an *Anopheles*-specific prey-capture tactic that enables it to exploit *Anopheles*' distinctive resting posture with its abdomen angled up from the substrate [21] (i.e., the spider moves behind and under the mosquito's abdomen and then attacks from below). As this tactic is not adopted by larger individuals, our initial expectation was that only small juveniles of this predator might prefer *Anopheles*. However, after modifying previous testing methods, a more complex preference profile has emerged. In particular, a 7-day pre-trial fast was part of the protocol in the previous prey-choice study [11], the rationale being to ensure that the spider would be responsive to prey-identification cues, but here, besides testing fasted spiders, we also test sated spiders (i.e., spiders provided with unlimited access to midges and blood-fed *Anopheles* on the day before testing).

## Results/Discussion

**Figure 2:**
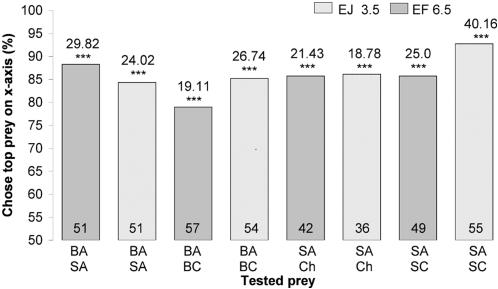
Simultaneous-presentation mount tests of sated *Evarcha culicivora.* Percentage: spiders that chose prey type at top list of two under bar. E = *Evarcha culicivora*. J = juvenile. F = adult female. BA = blood-fed *Anopheles*. SA = sugar-fed *Anopheles*. Ch = chironomid midge. BC = blood-fed *Culex*. SC = sugar-fed *Culex*. Spider body length in mm. N inside each bar. Tests of goodness of fit: chi-square statistic above bar; null hypothesis, 50/50; *** P<0.001.

First, using mount tests, we confirmed that sated spiders expressed the previously shown basic preference for blood-carrying mosquitoes (Fig. 2; BA, SA), but we also found that *E. culicivora* prefers *Anopheles* to another mosquito genus sympatric with it, *Culex*, with spider size and prior-feeding condition being important variables influencing this surprisingly specific preference.

**Figure 3:**
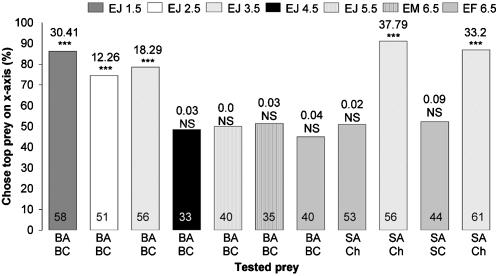
Simultaneous-presentation mount tests of fasted *Evarcha culicivora.* Percentage: spiders that chose prey type at top list of two under bar. E = *Evarcha culicivora*. J = juvenile. F = adult female. M = male. BA = blood-fed *Anopheles*. SA = sugar-fed *Anopheles*. BC = blood-fed *Culex*. Ch = chironomid midge. Spider body length in mm. N inside each bar. Tests of goodness of fit: chi-square statistic above bar; null hypothesis, 50/50; *** P<0.001.

**Figure 4:**
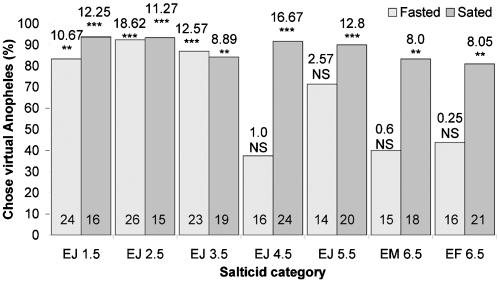
Simultaneous-presentation virtual-prey tests (blood-fed virtual mosquitoes in *Anopheles* rest posture and in *Culex* rest posture) of fasted and sated *Evarcha culicivora*. E = *Evarcha culicivora*. J = juvenile. F = adult female. M = male. Spider body length in mm. N inside each bar. Tests of goodness of fit: chi-square statistic above bar; null hypothesis, 50/50; *** P<0.001, ** P<0.01.

When fasted, small juveniles (≤3.5 mm in body length), but not the larger spiders, chose blood-fed *Anopheles* significantly more often than blood-fed *Culex*, regardless of whether they were tested with motionless mounts (Fig. 3) or with animated virtual mosquitoes (Fig. 4). There were no significant differences in how the different size sated spiders responded to lures made from these two mosquitoes (6.5 mm sated adult females, 45 of 57 chose blood-fed *Anopheles*; 3.5 mm sated juveniles, 46 of 54 chose blood-fed *Anopheles*) (Χ^2^_1_ = 0.7; NS). However, fasted juveniles chose blood-fed *Anopheles* significantly more often (44 of 56) than fasted adult females (18 of 40) (Χ^2^_1_ = 11.5; P = 0.001), and this same trend held when 3.5 mm juveniles and 6.5 mm adult females were tested with virtual mosquitoes. How often sated females chose virtual mosquitoes in an *Anopheles* posture instead of in the *Culex* posture (17 of 21) was not significantly different from how often sated juveniles chose virtual mosquitoes in the *Anopheles* posture (16 of 19) (Χ^2^_1_ = 0.1; NS). However, fasted juveniles chose virtual mosquitoes in *Anopheles* posture (20 of 23) significantly more often than adult females chose virtual mosquitoes in *Anopheles* posture (7 of 16) (Χ^2^_1_ = 8.3; P = 0.008).

Fasted juveniles, unlike fasted adult females, also chose sugar-fed *Anopheles* females (Fig. 3) more often than midges or sugar-fed *Culex* (all tests were with mounts). However, when sated, adult females as well as small juveniles chose blood-fed *Anopheles* significantly more often than blood-fed *Culex*, chose sugar-fed *Anopheles* significantly more often than sugar-fed *Culex*, and chose sugar-fed *Anopheles* significantly more often than midges (Fig. 2).

As mounts were motionless and virtual prey all moved identically, there were no potential movement cues by which spiders could discriminate between prey in mount tests or virtual-prey tests. The only variable in virtual-prey tests was prey posture. Yet even the smallest juveniles consistently chose *Anopheles*, indicating that the visual system of even the smallest juveniles (body length, 1.5 mm) have a remarkable capacity for identifying the preferred prey. We can not rule out the possibility that there were ultraviolet cues present when using living mosquitoes, and mounts, that were absent from the virtual mosquitoes. Jumping spiders have UV-sensitive receptors [16], [22], [23], [24], and UV-based signals may be used during salticid intraspecific interactions [25]. However, given the consistency of our findings, there is no suggestion that the presence-versus-absence of UV was an important variable in the present study.

When the alternative is a blood-fed *Culex*, perhaps the small spiders' preference for blood-fed *Anopheles* is not surprising, as this is consistent with small juveniles having *Anopheles*-specific prey-capture behavior [20], but large spiders can easily overpower mosquitoes without adopting the special tactic used by small juveniles. As might be expected, there was no evidence that large, fasted, spiders discriminated between blood-carrying *Anopheles* and *Culex*. However, small, fasted, juveniles apparently prefer *Anopheles* even when choosing does not provide a blood meal, and both large and small sated spiders appear to have an underlying preference for *Anopheles* independent of gaining blood meals.

It was only when we tested sated spiders that we showed *Anopheles* to be salient to large *E. culicivora* individuals. Whether the sated condition is common for *E. culicivora* in nature is unknown, but Lake Victoria is notorious for supporting enormous populations of midges [14], suggesting that, in nature, the sated condition simulated in our experiment is closer to the norm than the fasted condition.

**Figure 5:**
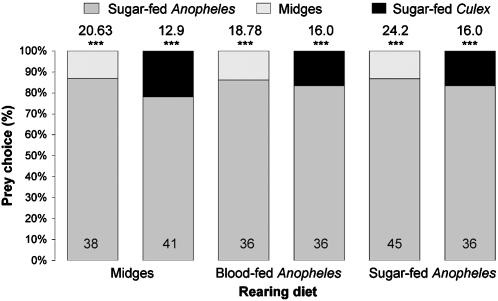
Simultaneous-presentation mount tests of sated *Evarcha culicivora* females (body length 6.5 mm) from cultures with non-standard feeding regimes (midges only, blood-fed *Anopheles* only and sugar-fed *Anopheles* only). N inside each bar. Tests of goodness of fit: chi-square statistic above bar; null hypothesis, 50/50; *** P<0.001.

For ascertaining whether diet influenced preference, we altered the feeding regime. Besides testing spiders from cultures maintained for two generations on the standard diet (blood-fed *Anopheles* plus midges), we also tested sated adult females from cultures maintained for two generations on midges only, blood-fed *Anopheles* only and sugar-fed *Anopheles* only and these spiders also chose sugar-fed *Anopheles* significantly more often than midges or sugar-fed *Culex* (Fig. 5). Evidently, preference for *Anopheles* is independent of prior experience with blood and with *Anopheles*.

This is the first demonstration of a spider, or any predator, singling out *Anopheles* mosquitoes as preferred prey. All active size classes of *E. culicivora* chose *Anopheles* in preference to other mosquitoes and other dipterans, but expression of this preference varied with spider size class and prior feeding condition, and *E. culicivora*'s expression of this predator's preference for *Anopheles* appears to be independent of gaining blood meals.

One of the implications of our findings is that predators may sometimes adopt surprisingly specific preferences and that even insects that adversely affect human health may be singled out by natural predators. Additional work is needed before we will understand how *E. culicivora* might benefit by choosing *Anopheles* and for determining whether this predator might have a role in efforts to control malaria.

## Materials and Methods

The field site and laboratory were at ICIPE's Thomas Odhiambo Campus (Mbita Point, Kenya). Each of the prey types that we used was sympatric with *E. culicivora* at Mbita Point. Basic experimental protocol and rearing procedure was as in earlier studies [11], [20], and only essential details are provided here.

Testing was carried out between 0800 h and 1100 h (laboratory photoperiod 12L∶12D, lights on at 0700). Midges were adult females of *Nilodorum brevibucca* and mosquitoes were adult females of either *Anopheles gambiae* s.s. (referred to simply as ‘*Anopheles*’) or *Culex quinquefasciatus* (‘*Culex*’). Each adult mosquito was from one of two groups, ‘blood-fed’ or ‘sugar-fed’, both of which were maintained on glucose (6% solution), but blood-fed mosquitoes also received human blood three times per week. Blood-fed mosquitoes received blood 4–5 h before becoming prey during rearing or being used for making mounts.

Discrete spider size classes (body length to nearest 0.5 mm) were used (measured with ocular micrometer) (juveniles: 1.5 mm, 2.5 mm, 3.5 mm, 4.5 mm, 5.5 mm; adult males and females, 6.5 mm) (‘small’, no larger than 3.5 mm; ‘large’, no smaller than 4.5 mm). Adult males and females matured 18–20 days before being tested and none had mated. All spiders came from laboratory cultures (F2 generation) and, unless stated otherwise, they were maintained through to the F2 generation on the ‘standard feeding regime’ (blood-fed *Anopheles* once a week and midges twice a week). The exceptions were spiders were fed on midges only, sugar-fed *Anopheles* only or blood-fed *Anopheles* only (culturing methods identical to the standard except for prey type).

Fasted spiders were kept without prey for 7 days before being tested. Sated spiders were provided with unlimited prey on the day before testing. For spiders from the standard feeding regime, this was achieved by putting three midges and three blood-fed *Anopheles* in each spider's cage, observing the spider throughout the day and maintaining this number and combination of prey by replacing any prey that were eaten or died of other causes. For spiders from the other three feeding regimes, sated spiders were maintained the day before testing with a replenishing supply of six individuals of the prescribed prey type.

Prey choice was ascertained in mount tests and in virtual-prey tests (Fig. 1) by simultaneous presentation of two prey types. As in the earlier study [11], mount tests were carried out using motionless lures (prey mounted in lifelike posture on cork disks) positioned outside a glass box, but visible to the spider inside. The spider made a ‘mount choice’ by entering and staying >30 s inside a glass tube that, by extruding from the box, led closer to one of the two mount types. In virtual-prey tests, spiders made a ‘virtual-prey choice’ by stalking one of two animated 3D drawings of mosquitoes and the only variable by which the two prey differed was posture (*Anopheles*: abdomen tilted up; *Culex*: abdomen horizontal).

For drawing 3D virtual mosquitoes, images were first captured (Zeiss AxioVision 3.1 software; resolution, 1300(h)×1030(v) pixels) from preserved blood-fed *Anopheles* specimens (adult females) using a stereomicroscope (Leica MZ12.5, with a Planapo 1.0× objective) and a digital camera (Zeiss AxioCam HRc CCD). From these images, virtual mosquitoes were drawn and animated using 3D Studio Max. Virtual mosquitoes had red abdomens and grayscale heads and thoraces. Each virtual-mosquito antenna was made by using a photograph of an antenna to surface a transparent ‘box’. By using ‘bend’ and ‘twist’ functions in the software, the virtual antenna was given 3D appearance.

A 10-s movie file, showing two virtual mosquitoes side by side, was programmed to loop continuously in a computer. The two virtual mosquitoes differed only in their posture, one in *Anopheles*' typical resting posture (body tilted 45°) and the other in *Culex*'s typical resting posture (body held parallel to the substrate [21]). Rendered movies (avi format) were forward-projected (800×600 pixels) on to a screen using a Telex P400 LCD data projector (frame rate of animation files 25 frames per second). The screen (fine-ground matte unmarked type D Nikon F3 focusing screen, 39 mm wide×30 mm high) was situated *c.* 150 mm from the projector lens, in front of which there was a ramp (stainless steel, 15 mm wide×150 mm long) (Fig. 1). The distance between the screen and the top end of the ramp was 2 mm when testing small spiders and 5 mm when testing large spiders. These screen-ramp distances ensured that spiders could not walk on to the screen (i.e., spiders that attacked virtual prey had to leap). The projector angled down by 10° and the screen sat in front of the top end of a stainless steel ramp, angling up by 25°. With this configuration, spiders walked up the ramp without entering the light path from the projector.

Large spiders were first taken into a transparent PVC tube (10 mm long; inner diameter 8 mm) and the two ends were plugged with corks. Using BluTac, the tube was held positioned along the midline of the ramp, oriented in the same direction as the ramp, with the closer end 50 mm from the top of the inclined ramp. After we removed the cork on the upward-facing tube end, testing began when the spider walked out of the tube and on to the ramp.

Preliminary trials revealed that this method was problematic when using small spiders because, at 50 mm away, small spiders often seemed not to notice the virtual mosquitoes and, when closer to the screen, the tube cast a shadow on to the screen. Our solution was first to entice a small spider on to the tip of a small soft paintbrush and then to touch the ramp with the tip of the brush 10 mm from ramp's upper end. Testing began when the small spider walked off the brush on to the ramp, facing the screen.

Which of the two virtual mosquitoes was on right side of screen was determined at random. The body length of virtual mosquitoes was 3.5 mm on the screen (image size reduced by a second lens in front of the projector lens). When displayed, the two virtual mosquitoes were side by side (10 mm apart) and they moved simultaneously in a way that simulated *Anopheles*' natural grooming behavior (groomed for 1 s;1-s interval between successive grooming bouts). Rendering of virtual grooming behavior was achieved by first video taping natural grooming by living *Anopheles* females and then, based on frame-by-frame analysis of the digital video footage, transferring typical grooming movement to the virtual mosquito.

Our criterion for recording a spider's ‘choice’ was met only when the spider stalked a virtual mosquito (i.e., with body lowered, the spider approached one of the two virtual mosquitoes while maintaining orientation of principal eyes toward this mosquito), reached the upper end of the ramp and either stopped and remained facing to this virtual mosquito for 30 s or else leapt on to it. Spiders were allowed 15 min to make a virtual-prey choice, except that, if the spider was stalking when the 15-min period ended, the test period was extended long enough for it complete the stalking bout. Testing was aborted whenever spiders leapt or ran (instead of walking calmly) on to the ramp, took longer than 10 min to move from a tube or a brush on to the ramp, remained on the top of the ramp, but failed to initiate a choice within 15 min, or left the top of the ramp before making a choice or before 15 min elapsed. Aborted tests were rare (<5%).

Our testing procedures and choice criteria controlled for how a living prey's reactions to the predator might have influenced test outcome. However, by adding another criterion before recording a spider's choice, we confirmed that each spider's decision during an encounter with mounts or virtual mosquitoes (i.e., surrogate prey it could not eat) revealed actual motivation to eat the chosen prey. This was achieved by submitting each spider to a live-prey test on the day following a successful mount or virtual-prey test. In live-prey tests (methods same as in an earlier study; 26), two living prey of the same types as used in a mount test or in a virtual-prey test on the previous day were introduced into the spider's cage. A spider's ‘live-prey choice’ was the first prey eaten. Our criteria for recording that a spider ‘chose’ included a requirement that the spider's live-prey choice matched its mount or virtual-prey choice. Regardless of the testing procedure (live, mount or virtual-prey tests), one of the two prey types was always *Anopheles*.

No spider or mount was used in more than one test. The body length of all mounted insects was 4.5 mm. Sample sizes for mount tests and virtual-prey tests were always 70 and 30 spiders, respectively (note, however, that we used data from only the tests during which the spider made the same choice during the following live-prey testing). Results were analyzed using chi-square tests for goodness of fit (null hypothesis: equally likely to choose each of the two prey types) and chi-square tests of independence.
